# Implant rehabilitation of edentulous maxilla in digital dentistry: A case report utilizing CAD/CAM technologies

**DOI:** 10.34172/joddd.2021.020

**Published:** 2021-05-05

**Authors:** Victor Haruo Matsubara, Amit Prem Gurbuxani, Sammy Francis, Robert J Childs

**Affiliations:** ^1^Department of Restorative Dentistry, Faculty of Health and Medical Sciences, Dental School, University of Western Australia. Perth, Western Australia, Australia; ^2^Department of Periodontics, Faculty of Health and Medical Sciences, Dental School, University of Western Australia. Perth, Western Australia, Australia

**Keywords:** Dental prosthesis, Maxilla, Aesthetics, Dental implants, Computer-aided design, Computer aided manufacturing

## Abstract

The replacement of missing teeth utilizing dental implants and digital dental technologies has gained significant popularity in daily clinical practice over the last decade. Partially dentate patients present more anatomical references to guide the implant position and prosthetic reconstruction as compared to completely edentulous arches. Therefore, the management of edentulous maxilla using implant digital dentistry represents a challenging clinical situation where a thorough treatment plan is paramount to achieve a final prosthetic result that meets both functional and esthetic requirements. This case report discusses the oral rehabilitation of an edentulous maxilla and partially dentate mandible using a *digital workflow* for both the *surgical and prosthetic* phases of the implant therapy. Protocols for clinical assessment, treatment planning, and restorative management are described to provide a predictable and prosthetic-driven treatment for implant-supported prostheses.

## Introduction

Tooth loss has a negative impact on oral function, thus resulting in decreased quality of life of edentulous patients and increased risk of early mortality.^1^ Aiming to reestablish esthetics and oral function, the fabrication of complete dentures has been the first choice of prosthetic treatment for many years. However, the predictability and success of full-arch osseointegrated implant rehabilitation initiated a new era in the management of edentulous patients.^2^

As edentulism often results in advanced atrophy of the residual alveolar bone and loss of facial support, the prosthetic rehabilitation of an edentulous maxilla using implants usually involves a complex scenario, where multiple variables need to be considered when planning and executing treatment.^3^ Hence, a prosthetic-driven approach and careful consideration of the plan are paramount to achieve esthetics and functional outcomes that fulfill both patient and clinical expectations.

The success of prosthetic treatment relies not only on the clinical skills and knowledge but also depends on the quality of laboratory work.^4^ Therefore, inefficient communication and cooperation between dentist and dental technician may lead to unsuccessful treatment outcomes. Digital dentistry facilitated this relationship by allowing a more precise acquisition and transmission of patient information that, in turn, results in more predictable implant therapy.^5^ Implant guided surgery illustrates very well this advancement, as the ideal implant position is established in a virtual implant planning software based on both computed tomography images and a virtual prosthesis. This digital approach provides predictability during the implant placement and prosthetic reconstruction phase.

As a result of recent improvements in intraoral digital scanners, digital dental software, computer-aided design and computer-aided manufacturing (CAD/CAM) technology, and imaging tools such as cone-beam computed tomography (CBCT), it is now possible to integrate different types of digital files into the same CAD software. Therefore, the clinical and laboratory steps of full-arch implant treatment have been changing continuously to integrate different digital tools that make the prosthodontic treatment more time and cost-efficient.^6,7^

The description of mandibular rehabilitation using implants and digital tools has been vastly explored in the literature,^8,9^ however, the fabrication of full-arch implant-supported fixed maxillary prosthesis is yet considered a gray area in digital dentistry with limited number of publications showing predictable management of the case.^10^ This disparity between mandible and maxilla might be explained by the fact that the edentulous maxillary arch represents a more challenging scenario as compared to the mandibular arch due to its aesthetic demand, which involves facial support as well as dental and gingival visibility factors.^3^ As a result, many clinicians are hesitant to migrate to digital workflows when dealing with implant-prosthetic rehabilitation of edentulous maxilla, especially when natural teeth are still present.

Hence, this case report aims to describe a predictable digital workflow for the fabrication of a CAD/CAM maxillary full-arch implant-supported prosthesis in a patient with compromise maxillary dentition and show the importance of integrating cutting edge-technologies and conventional prosthetic procedures to achieve a maxillary fixed prosthesis that meets all the functional and aesthetic requirements.

## Case Report

**Figure 1:**
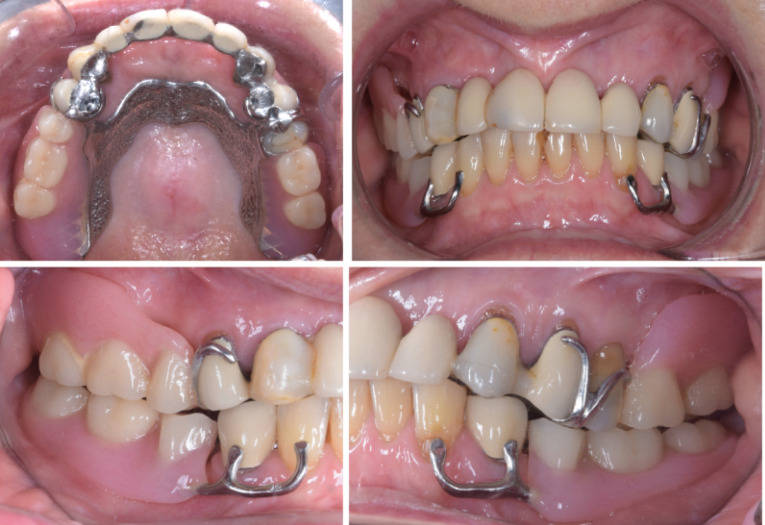
Presenting condition of the patient. Long span metal-ceramic bridge replacing all missing anterior maxillary incisors and supported by canines and first premolars. Cobalt-chrome removable partial denture replacing missing posterior maxillary and mandibular teeth.

A 79-year-old Caucasian woman in good systemic health presented to the clinic reporting “sore gums” in the maxillary right canine region. After a thorough clinical examination, radiographs, diagnostic casts, and diagnostic articulator mounting, the tooth 13 was given hopeless prognosis as an abutment tooth required to support the anterior maxillary ceramic fixed dental prosthesis (Figure 1). The same abutment tooth was involved in supporting a Kennedy class I removable partial denture. Considering the loss of strategic teeth and the patient’s desire of having fixed prostheses, extraction of all maxillary teeth followed by the placement of six implants using a computer-guided surgical approach to support a screw-retained full-arch ceramic prosthesis was planned. In the mandible, the patient was wearing an unsatisfactory Kennedy class I removable partial denture with poor retention and uneven occlusal plane. The curve of Spee was inverted at premolar area with overeruption of maxillary premolars, especially on the left-hand side. The mandibular first premolars had grade II mobility and horizontal bone loss, whereas the anterior teeth were vital with only minor incisal wear. Mandibular implant-supported fixed partial dentures were included in the treatment plan to replace the existing removable partial denture.

This patient had a favorable medical history with no contraindications for surgical procedures and her plaque control was conducive to implant therapy. All extra- and intra-oral factors were assessed during the clinical examination, and they all showed favorable condition for a fixed implant-supported prosthesis, including a low smile line and sufficient lip support. As the patient did not have significant bone loss in the anterior maxilla, the absence of a buccal flange in the definitive prosthesis would not compromise the lip support and the lower facial third aesthetics. An informed consent from the patient was obtained before commencing treatment.

Aiming at safe and predictable implant therapy, the first part of treatment involved the atraumatic extraction of all maxillary teeth and insertion of an immediate complete denture as an interim prosthesis. Using a semi-adjustable articulator, the tooth setting of the denture was established to accommodate optimal esthetic and occlusal requirements (bilateral balanced occlusion), including midline position, lip support, anterior tooth display, parallelism between incisal edges and interpupillary line, occlusal vertical dimension (OVD) and both curve of Spee and curve of Wilson. As the patient had no temporomandibular joint disorder symptoms and adequate facial support, the immediate denture followed the existing OVD and centric occlusion with a bilaterally balanced occlusal scheme. A mandibular acrylic partial denture was also fabricated to provide adequate occlusal contacts after the extraction of the mandibular premolars that were periodontally compromised.

**Figure 2:**
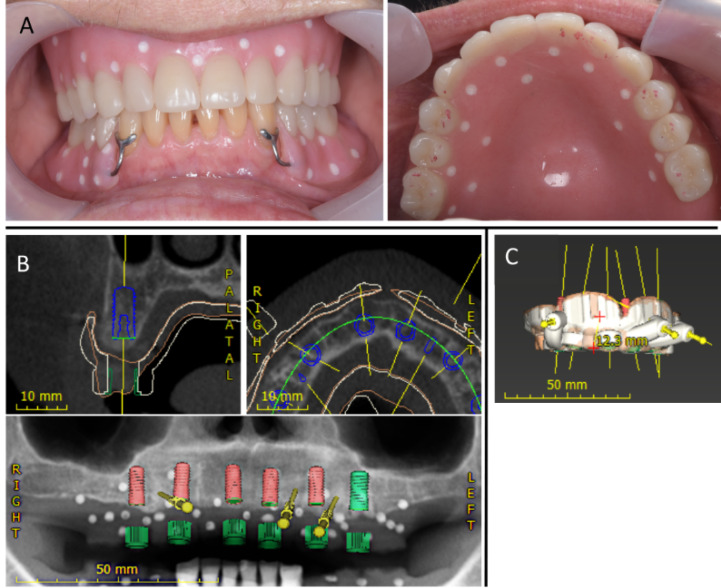
(A) Immediate maxillary denture and a new mandibular acrylic partial denture with radiopaque markers that were used to guide the digital implant planning. (B) Digital implant planning using coDiagnostiX software based on CBCT images to orientate the position of the implants. Six implants were planned to be placed in the incisor and premolar regions. (C) Surgical guide was designed using the same software.

**Figure 3:**
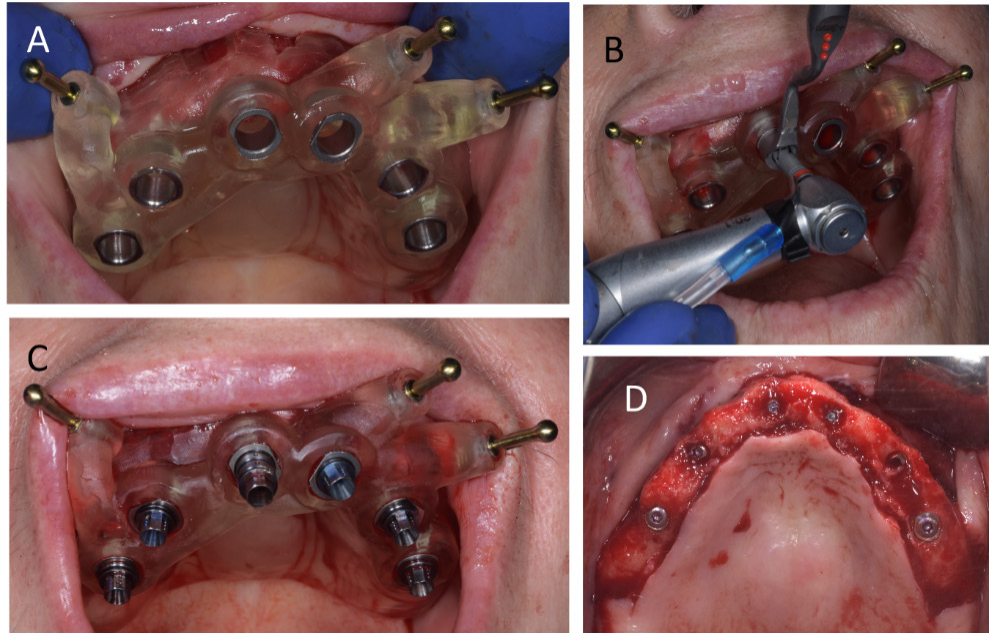
Open flap fully guided implant surgery sequence. (A) 3D printed surgical guide stabilized in the maxilla by three anchor pins. A putty key was used to stabilize the surgical guide before its fixation. (B) Surgical sleeves guided the drilling procedures. (C) Six Straumann BL implants were placed guided by the surgical guide. (D) Alveolar ridge regularization with implants placed as planned digitally.

After a four-month healing period, CBCT images were used for the digital implant planning. Radiopaque markers were added to the maxillary and mandibular acrylic dentures for reference purposes, and the CBCT scan was taken with the patient wearing both upper and lower dentures (Figure 2A). Using an implant planning software (coDiagnostiX, Dental Wings Inc., Montreal, Canada), the ideal location of the implants was established according to the denture teeth position and the available bone (Figure 2B), thus respecting a prosthetic-driven approach for the treatment plan. The surgical guide was designed using coDiagnostiX software (Figure 2C) and 3D printed using an SLA desktop 3D printer (Form 2, Formlab, USA). After removing the supporting structure and further refinement, the sleeves for fully guided surgery were inserted in the guide.

During the digital implant planning phase, it was noted that the edentulous alveolar ridge would need regularization as the uneven residual bone could affect the soft tissue contour negatively under the future implant-supported prosthesis. In this case, the adjustment of crestal bone levels would not only allow the placement of implants in the ideal vertical position but also facilitate the access for adequate plaque control under the fixed prosthesis and peri-implant tissues.

**Figure 4:**
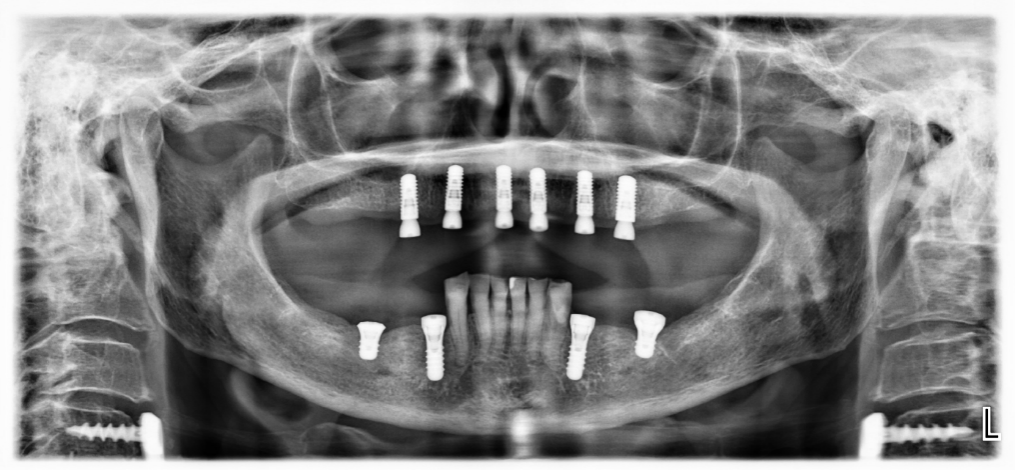
Panoramic radiograph after osseointegration of implants.

Six implants (five 4.1 x 10 mm and one 4.8 x 10 mm Straumann SLA Bone Level implants) were planned to be evenly distributed in the maxilla so as to minimize the extension of posterior cantilevers, using guided surgery (Figure 3). Open flap surgery was necessary as the regularization of the edentulous bone would be necessary prior to the implant placement. In the mandible, four Straumann tissue level implants were placed bilaterally to support two ceramic fixed partial dentures (Figure 4).

After osseointegration, healing abutments were inserted and a maxillary midline frenectomy was performed in the same surgical procedure to treat a high attached frenum, which may interfere with the placement of the fixed prosthesis. The maxillary denture had to be relined with soft material (Soft-liner, GC, Sydney, AU) due to significant changes in the alveolar ridge contour after the soft tissue healing.

After the healing period of the second-stage surgery, conventional implant impression was performed using an open custom tray approach with polyvinyl siloxane (PVS - Aquasil Ultra LV, Dentsply Caulk, Milford, DE) as the impression material. A fixture level impression was performed with the impression abutment splinted together using light-cured resin. The corresponding implant lab analogs were attached to the impression abutments and the master cast was fabricated in a conventional fashion.

In the same appointment, the immediate complete denture was duplicated in acrylic resin and this copy denture was “relined” with the same impression material used for the implant impression. Care was taken to maintain the same OVD and horizontal jaw relation during this clinical step. The impression of opposing arch was taken with the mandibular acrylic partial denture in position to produce the opposing model. Both maxillary and mandibular models and the copy denture were sent to the dental laboratory for scanning.

In the laboratory, the screw-retained abutments were selected according to the implant angulation and mucosa thickness in the model. Straumann CARES^®^ Mono Scanbodies for screw-retained abutments were used to scan the maxillary master model and create a digital model. Both master model and copy denture were scanned using a laboratory scanner (Straumann^®^ CARES® 7 series scanner) and all scan data were sent to a CAD software (DentalCAD, Exocad, Germany).

**Figure 5:**
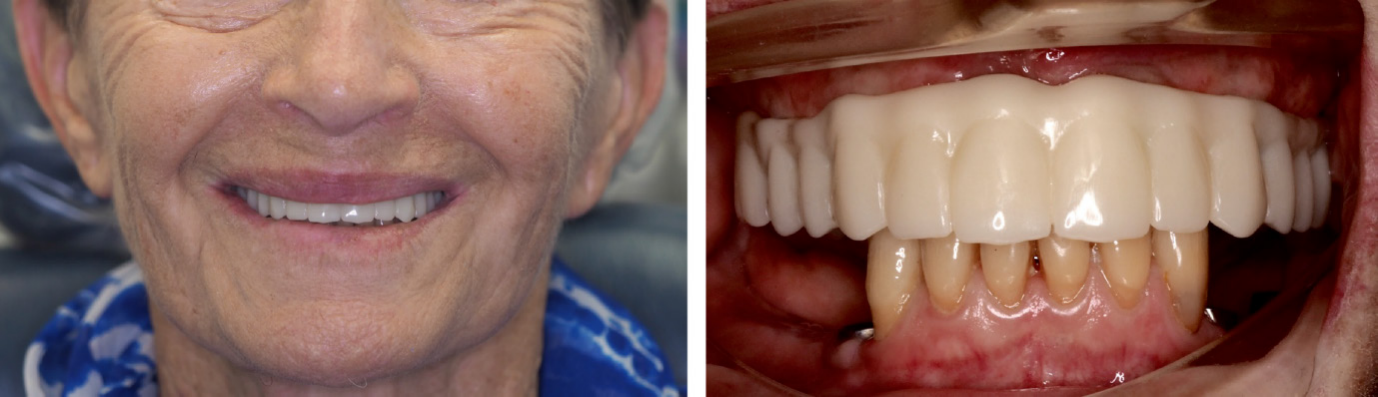
PMMA try-in prototype was used for four weeks. Minor corrections in the curve of Spee, buccal corridor and length of canines were found necessary (images taken before adjustments done).

The digital images provided information about implant location, soft tissue contour, ideal position of the denture teeth, and vertical and horizontal maxillo-mandibular relations (OVD and centric relation). Based on this digital data, a CAD-CAM screw-retained try-in prototype was milled in tooth-colored polymethyl methacrylate (Aidite Multilayer, Aidite Technology Co, China), and connected to the implants using temporary titanium copings (Figure 5).

The prototype was inserted and its passive fit was checked. The prototype was adjusted according to esthetic appearance and occlusal contacts (centric and dynamic). The passive fit of the try-in denture was confirmed clinically and radiographically. An implant verification jig was fabricated with self-curing Pattern Resin (GC, Sydney, AU) to verify the accuracy of the master model prior to manufacturing the zirconia framework.

Minor corrections in the curve of Spee, buccal corridor and length of canines of the try-in prototype were necessary to attain ideal occlusal and aesthetics requirements. The gingival and flange contour was adjusted to create a convex surface that facilitate daily plaque control under the fixed prosthesis. The patient was allowed to wear the prototype as a temporary implant-supported prosthesis to assess the masticatory function and speech.

**Figure 6:**
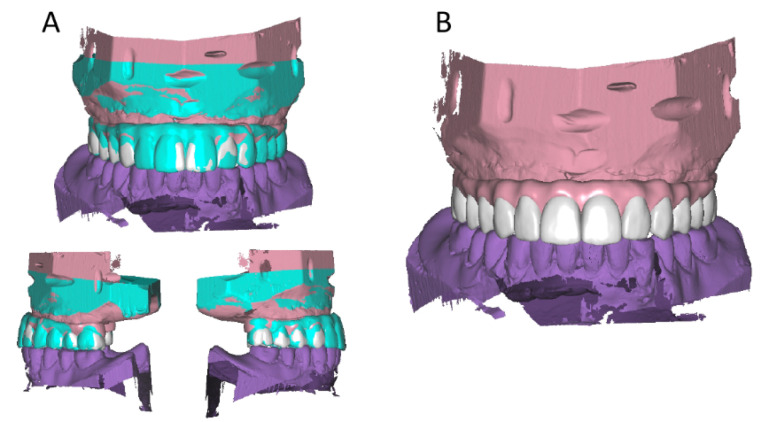
(A) The design of the definitive implant-supported prosthesis was based on the try-in prototype (green) that was scanned after the adjustments in the patient’s mouth. (B) Final design of the implant- supported prosthesis.

After four weeks wearing the prototype, it was confirmed that the patient was satisfied with the oral functions (masticatory and phonetic) and the aesthetics of the prototype and was able to keep a good plaque control in the maxillary arch. The acrylic prototype was sent to the laboratory together with a bite record in centric (O-Bite, DMG, Germany) to be scanned as a pre-op model. This digital model was used as a more accurate reference for the final prosthesis. Using the Exocad Dental CAD software, the definitive ceramic prosthesis was designed following the contour of the try-in prosthesis (Figure 6).

**Figure 7:**
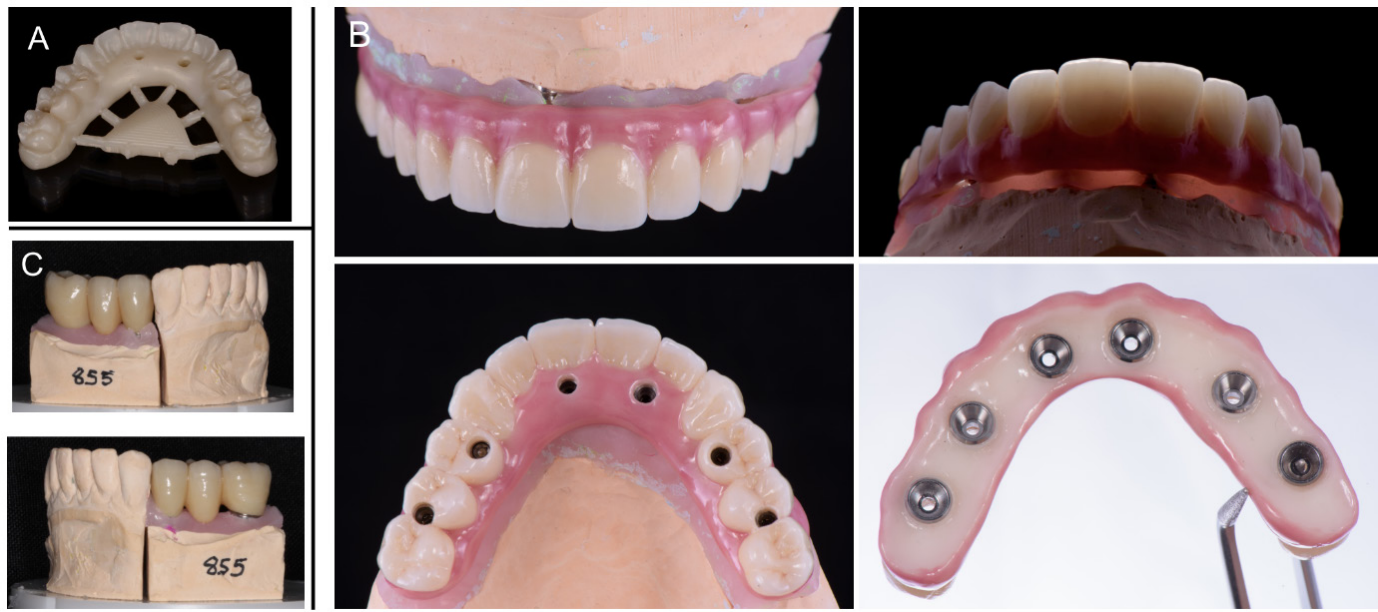
(A) Zirconia framework milled in highly translucent zirconium oxide with buccal/labial cutback. Occlusal and palatal surfaces were left in zirconia. (B) Final veneering technique with the application of a layering ceramic on the buccal/labial surfaces, associated with the application of pink stain on the surfaces corresponding to soft tissues. (C) All-ceramic screw-retained prostheses in the mandibular arch.

Aiming for a prosthetic device with favorable mechanical properties and outstanding aesthetics, a full-arch porcelain-veneered zirconia prosthesis was fabricated for the maxilla. The zirconia framework was milled in highly translucent zirconium oxide (Ceramill Zolid HT, Amann Girrbach AG, Germany) with monolithic palatal and bucco-labial flanges. Only the bucco/labial surface of the teeth themselves were designed and milled with a cutback for ceramic layering (Figure 7A). The final milled zirconia framework was sintered overnight, trimmed and adjusted to the final shape. The final veneering technique required the application of a layering ceramic (IPS e.max Ceram ZirLiner, Ivoclar Vivadent, Australia). Tooth-colored porcelain was applied concurrently with pink stain GC Initial Lustre paste (GC, Sydney, Australia) on the flanges followed by glazing (Figure 7B). The final prosthesis was cemented onto Straumann Variobase® copings for screw-retained abutments using resin cement (G-CEM Linkforce, GC, Sydney, Australia).

**Figure 8:**
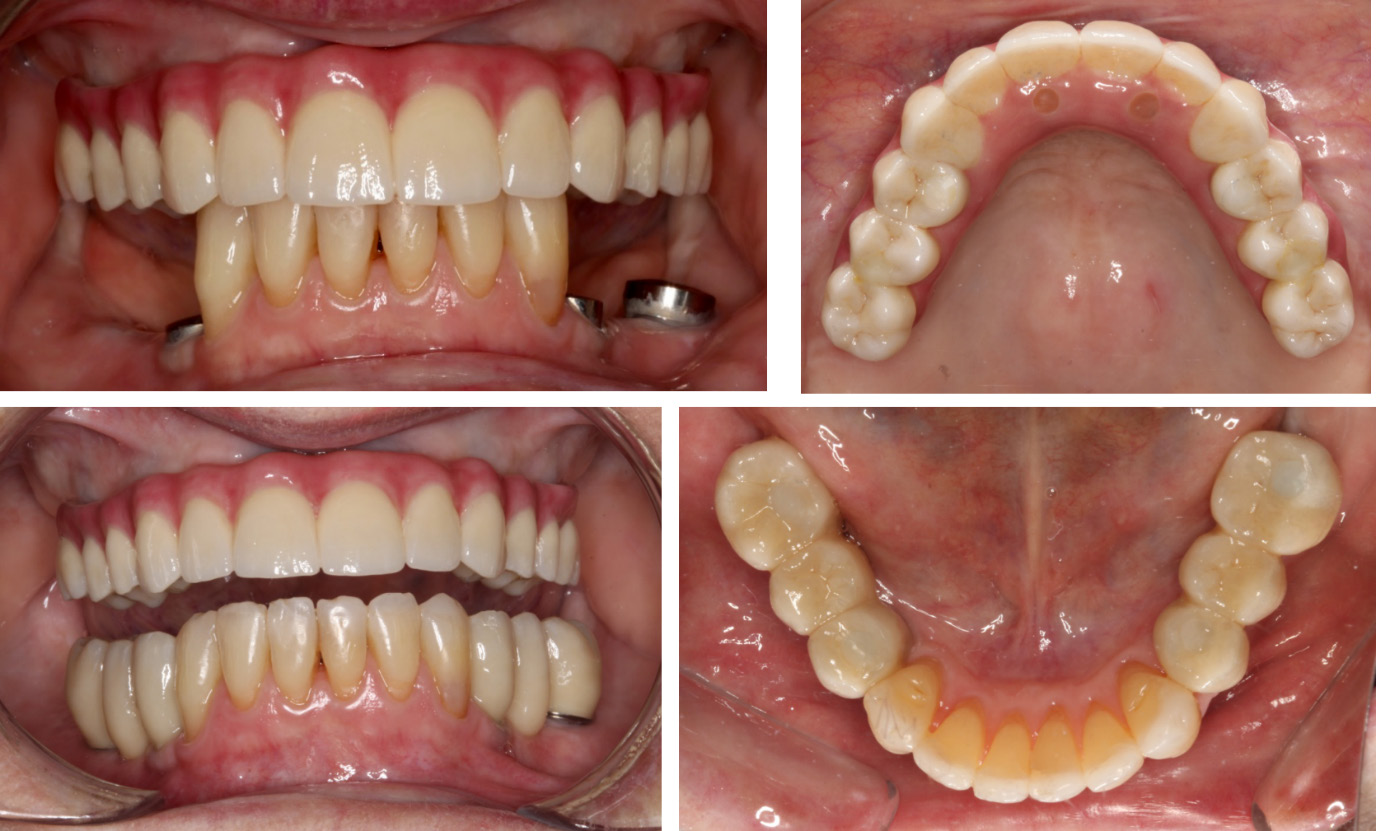
Final maxillary and mandibular all-ceramic screw-retained prostheses.

Screw-retained abutments were tightened before the installation of the definitive screw-retained prosthesis according to the manufacturer’s recommendation. Screw-access channels were sealed and restored using dental polytetrafluoroethylene (Teflon) tape and composite resin (Figure 8).

The mandibular implant-supported fixed partial dentures were fabricated following similar fabrication steps concomitantly to the fabrication of the maxillary prosthesis. The impression technique also used open custom tray with implant abutments joined with light-cured resin to prevent distortions. The incisal edge anatomy of anterior teeth was reestablished with direct composite restorations before taking the final impression. These restorations increased the OVD slightly without compromising comfort and function. The mandibular prostheses were also milled in highly translucent zirconium oxide and layered with tooth-color porcelain (Figure 7C). However, these differed from the maxillary prosthesis in that the occlusal surfaces were in zirconia, and the mandibular fixed partial dentures had the occlusal surfaces in a layering ceramic (IPS e.max Ceram ZirLiner, Ivoclar Vivadent, Australia) in order to create a softer occlusal contact surface (Figure 8) as zirconia tends to wear more the opposing tooth.^11^

## Discussion

The rehabilitation of edentulous maxilla using implants is often challenging even for experienced clinicians due to both surgical and prosthetic factors that influence clinical decision-making. Using a multidisciplinary approach, it was defined that the ideal moment for the implant placement in this case would be after hard and soft tissue healing. A stable alveolar bone would provide a more predictable reference for implant digital planning, thus avoiding undesirable implant surface exposure after osseointegration.

Different implant surgical approaches were considered for the case with variation in the number, size, and position of implants. One option available was the “All-on-Four” technique that uses four implants with the most distal ones tilted to avoid bone grafting in the edentulous maxilla.^12^ Despite the high rate of implant and prosthesis success of “All-on-Four” within five years,^13^ the loss of one implant would represent the failure of the treatment as a minimum of four implants is necessary for a fixed implant-supported prosthesis. In turn, the Brånemark protocol for the rehabilitation of edentulous maxilla suggests the use of five or six implants distributed in the anterior and posterior region of the maxilla to support a fixed prosthesis.^14^ According to the literature, placing six or more implants will incorporate better stress distribution, and avoid long cantilevers and redundancy of implant support, which prevents prosthesis loss if a single implant is lost.^10^ Therefore, aiming for a long-lasting implant-supported prosthesis, it was decided to place six implants parallel to each other in the maxilla.

Using a shortened dental arch approach,^15^ it was possible to distribute all implants in the maxillary incisor and premolar regions and avoid grafting procedures or non-grafting approaches utilizing short or tilted implants. The reduction of cantilever extensions in a shortened dental arch with implants placed anteriorly to the maxillary sinus would provide favorable stress distribution.^16^ The guided implant surgery approach also added extra predictability and safety to the treatment, avoiding complications such as mal-aligned implants and perforation of the maxillary sinus.

In the prosthetic phase, the splinted impression technique was adopted, using PVS impression material and open-custom tray, which is considered a reliable and accurate technique when treating fully edentulous patients.^17^ Although the digital impression is gaining increasing popularity, the literature does not recommend its use for full-arch cases as yet, due to less accuracy and local deviations of the full-arch digital impression as compared to conventional impression methods.^17,18^ The risk of distortion and inaccuracy of the digital impression can be even higher for intra-oral edentulous arch scanning considering that there are no teeth to provide points of reference for the scanning process. Despite the current limitation for full-arch treatment, the intraoral digital scanning is considered a clinically acceptable alternative in the fabrication of single crowns and short fixed dental prosthesis.^18^

The final maxillary screw-retained ceramic prosthesis was designed to meet all the esthetic, functional and hygienic requirements, being comfortable and easy to clean. As two types of interim prostheses were used during the treatment (immediate complete denture and screw-retained acrylic prosthesis), it was possible to identify and correct occlusal and aesthetic errors before the fabrication of the final prosthesis. Consequently, adjustments on the zirconia surface were not needed after the prosthesis installation, thus avoiding the introduction of micro-cracks in the ceramic that may propagate and cause catastrophic fractures under loading.

All implant-supported prostheses of this case were screw-retained rather than cemented. The well-known benefits of screw retention, particularly being retrievability and the avoidance of excess cement that may lead to peri-implant diseases, supported this clinical choice.^19^ When dealing with extensive restorations, such as a full-arch maxillary prosthesis, the possibility of removing the prosthetic device for cleaning or repairing if necessary, without damaging the ceramic, makes the screw retention choice, the primary option when restoring implants.^20^

## Conclusion

**Figure 9:**
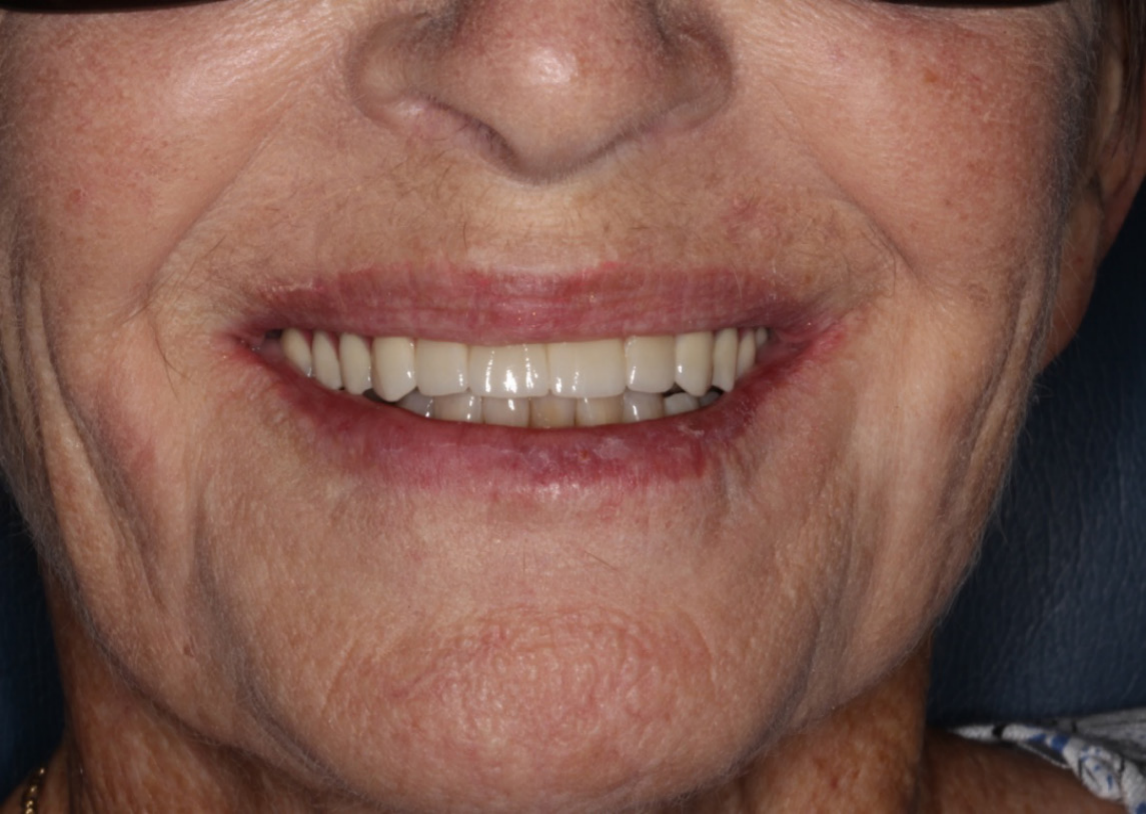
Six-month postoperative smile – anterior view.

This case report describes the oral rehabilitation of a patient with a compromised dentition and missing mandibular and maxillary teeth who wanted to improve comfort, masticatory function and aesthetics. A maxillary full-arch fixed ceramic prosthesis and mandibular ceramic fixed partial dentures, all supported by implants placed via guided surgery, were used to replace the missing teeth in a predictable manner. The planning and delivery of the treatment was facilitated and optimized with the aid of digital technologies. All the steps were planned to minimize the risk of errors and complications during both the implant placement and fabrication of the final prostheses, thus reducing the numbers of clinical sessions and the cost associated with repetitions. Hence, the success of the case (Figure 9) shows that a thorough clinical examination guiding the treatment plan, followed by the combination of digital and conventional workflows that facilitate the communication between clinician and dental technician, provide a predictable treatment outcome in implant rehabilitation of the edentulous maxilla.

## Authors’ contribution

VHM and APM : Study conception, prosthetic treatment delivery and drafting of manuscript

SF: Surgical treatment delivery and RJC: Revision of manuscript.

## Acknowledgement

The authors would like to acknowledge Mark Dallamora and Mijin Kim (Aesthetika) for their knowledge, technical expertise and artistry in the laboratory phases involved in this case, Joe Stokes and Jemma Morgan from Straumann for providing all the implants and prosthetic components.

## Competing Interests

None.

## Funding

None.

## Ethics Approval

The informed consent was obtained from the patient.
